# In Vitro Investigations of miR-33a Expression in Estrogen Receptor-Targeting Therapies in Breast Cancer Cells

**DOI:** 10.3390/cancers13215322

**Published:** 2021-10-23

**Authors:** Pelin Ozfiliz-Kilbas, Ozlem Sonmez, Pinar Obakan-Yerlikaya, Ajda Coker-Gurkan, Narcin Palavan-Ünsal, Pinar Uysal-Onganer, Elif Damla Arisan

**Affiliations:** 1 Department of Molecular Biology and Genetics, Istanbul Kultur University, Istanbul 34158, Turkey; p.ozfiliz@iku.edu.tr (P.O.-K.); sonmezzozlem@gmail.com (O.S.); 2 Department of Biomedical Engineering, Biruni University, Istanbul 34010, Turkey; pyerlikaya@biruni.edu.tr; 3 Department of Molecular Biology and Genetics, Biruni University, Istanbul 34010, Turkey; agurkan@biruni.edu.tr; 4 Department of Engineering, Netkent Mediterranean Research and Science University, 38-44 Kyrenia, Macka 99300, Turkey; narcinunsal@netkent.edu.tr; 5 Cancer Research Group, School of Life Sciences, University of Westminster, London W1W 6UW, UK; 6 Institute of Biotechnology, Gebze Technical University, Gebze 41400, Turkey

**Keywords:** estrogen, breast cancer, miR-33a, adipogenesis, FASN, fulvestrant

## Abstract

**Simple Summary:**

Altered metabolic pathways determine the aggressivity of breast cancer cells. To highlight the potential markers gains importance to understand early molecular signatures of disease. microRNAs are the small non-coding RNAs found in different biological samples. Due to the dysregulation of metabolic pathways, the expression and secretion of microRNAs are modulated.

**Abstract:**

(1) Background: Increased fatty acid synthesis leads to the aggressive phenotype of breast cancer and renders efficiency of therapeutics. Regulatory microRNAs (miRNAs) on lipid biosynthesis pathways as miR-33a have potential to clarify the exact mechanism. (2) Methods: We determined miR-33a expression levels following exposure of MCF-7 and MDA-MB-231 breast cancer cells to estrogen receptor (ER) activator (estradiol-17β, E2) or anti-estrogens (ICI 182,780, Fulvestrant, FUL) at non-cytotoxic concentrations. We related miR-33a expression levels in the cells to cellular lipid biosynthesis-related pathways through immunoblotting. (3) Results: miR-33a mimic treatment led to significantly downregulation of fatty acid synthase (FASN) in MCF-7 cells but not in MDA-MB-231 cells in the presence of estradiol-17β (E2) or Fulvestrant (FUL). In contrast to the miR-33a inhibitor effect, miR-33a mimic co-transfection with E2 or FUL led to diminished AMP-activated protein kinase α (AMPKα) activity in MCF-7 cells. E2 increases FASN levels in MDA-MB-231 cells regardless of miR-33a cellular levels. miR-33a inhibitor co-treatment suppressed E2-mediated AMPKα activity in MDA-MB-231 cells. (4) Conclusions: The cellular expression levels of miR-33a are critical to understanding differential responses which include cellular energy sensors such as AMPKα activation status in breast cancer cells.

## 1. Introduction

Breast cancer is the most frequently diagnosed malignancy in women [1]. Although associations between etiological factors and increased cancer risk have been shown to be variable through molecular subtypes of malignancies, a deeper understanding of risk factors is still needed. Epidemiological studies so far include obesity, high body mass index (BMI), metabolic syndrome, alcohol, and hypercholesterolemia as risk factors for breast cancer [2,3]. It is well clarified that dysregulation of fatty acid metabolism and lipogenesis leads to poor progression of breast cancer [4]. Moreover, fatty acid synthesis is linked with clinically aggressive tumor progression and rapid cell proliferation. Therefore, reducing tumor cell proliferation and increasing their apoptotic ratio are critical targets for improved treatment strategies [5]. The basic molecular mechanisms behind lipogenesis and lipid oxidation pathways are partly clarified by how to associate poor tumor progression, however, the underlying mechanisms of altered de novo fatty acid synthesis still remain unclear. Recent attempts show that targeting fatty acid synthase (FASN), a key enzyme that catalyzes the synthesis of endogenous long-chain fatty acid, could be a critical target for drug discovery. Moreover, sterol regulatory element-binding proteins (SREBPs) are master transcriptional regulators for de novo lipogenesis via increasing fatty acid and cholesterol synthesis, altering glycolysis pathways, and affecting uptake pathways [6,7]. Recent studies indicated that diet-induced obesity triggered expression levels of SREBPs in different malignant cells [8,9]. SREBP1 and SREBP2 are the isoforms which possess critical regulatory roles in fatty acid metabolism. SREBP1 has a role in the induction of fatty acid biosynthesis and triggers cell survival [7]. SREBP2 exists as an inactive precursor and is located in the endoplasmic reticulum [8]. According to the depletion of cholesterol levels, SREBP2 is cleaved, becomes activated, and translocated into the nucleus to upregulate genes that have roles in cholesterol uptake and biosynthesis and enhanced cholesterol levels [10]. Therefore, investigating the regulation of SREBP families could be important to overcome these issues [10].

MicroRNAs (miRNAs) are short (21–24 nucleotide long) non-coding RNAs that have roles in regulating gene expression. They can bind 3′ untranslated regions of mRNAs and lead to their inactivation or degradation [11]. Almost 50% of miRNA-encoding genes are located in the cancer-linked regions or fragile chromosomal sites [11]. It is well established that breast cancer cells display differential expression for different miRNA targets, which possess oncogenic and tumor suppressor roles compared to normal cells [12]. Therefore, the identification of disease-related miRNA targets is important for diagnosis and monitoring of disease progression. Additionally, significantly altered miRNAs are becoming important novel therapeutic tools to increase therapeutic success [13]. Related to lipid synthesis and metabolic mechanisms in the cells, previous studies showed that SREBP2 intron contains highly conserved miR-33a and SREBP1 intron contains miR-33b, which have roles in the regulation of cholesterol export and fatty acid oxidation [14]. Experimental evidence showed that miR-33a suppresses fatty acid biosynthesis enzymes and the insulin pathway, and therefore it is a critical molecule in regulating cholesterol metabolism. In addition, miR-33a was shown as a significant molecule in targeting cholesterol metabolism in cancer and might have a tumor suppressor role in the development of breast cancer [15]. Silencing of miR-33a enhanced plasma high-density lipoprotein (HDL) cholesterol and miR-33a deprivation caused enhanced serum HDL cholesterol levels in in vivo mouse models [16]. Moreover, miR-33a regulates AMPKα, ATPase, aminophospholipid transporter, class I, type 8B, member 1 (ATP8B1), hydroxyacyl-CoA dehydrogenase/3-ketoacyl-CoA thiolase/enoyl-CoA hydratase (trifunctional protein), beta subunit (HADHB), carnitine palmitoyltransferase 1A (CPT1A), sirtuin 6 (SIRT6), glypican 6 (GPC6), and phosphoenolpyruvate carboxykinase 1 (PCK1) and SREBP1 and SREBP2 [17]. In addition, anti-miR33 treatment was shown to decrease SREBP-1 expression levels and AMPKα levels and anti-miR-33a treatment regulated HDL cholesterol levels [18].

It is therefore critical to elucidate the potential role of miR-33a in fatty acid metabolism in breast cancer and to gain more information for developing new methodologies for clinical and therapeutic applications based on the ER status. For this purpose, we used two different breast cancer cell lines. We developed ER-deprived MCF-7 cells and identified the expression level of miR-33a in MCF-7 breast cancer cells in ER activation by ER agonist (estradiol-17β) and in ER deprivation using anti-estrogens (ICI 182,780, Fulvestrant). Estrogen-independent MDA-MB-231 breast cancer cells were also used for comparison to understand the potential role of estrogens in lipid synthesis and miR-33a-mediated responses.

## 2. Materials and Methods

### 2.1. Cell Culture

MCF-7 (HTB-22, ATCC, Manassas, VA, USA) and MDA-MB-231 (HTB-26, ATCC, Manassas, VA, USA) breast cancer cells were maintained in Dulbecco’s modified Eagle’s medium (PAN Biotech, Aidenbach, Germany, DMEM) supplemented with 10% fetal bovine serum (PAN Biotech, Aidenbach, Germany, FBS), 2 mM L-Glutamine (PAN Biotech, Aidenbach, Germany), 100 IU penicillin, and 100 μg/mL streptomycin (PAN Biotech, Aidenbach, Germany). Cells were grown at 37 °C in a humidified 5% CO_2_ incubator (Biological Industries, Kibbutz Beit-Haemek, Israel). 17-β-Estradiol (E2, activation of estrogen, 10 mM stock concentration) and Fulvestrant (FUL, inhibition of estrogen 10 mM stock concentration) were prepared (Selleckchem, Pittsburgh, PA, USA). 100 nM E2 and 500 nM FUL were used in further studies.

### 2.2. Cell Transfection

Syn-hsa-miR-33a miRNA mimic and anti-has-miR33a miRNA inhibitor were performed at 20 µM stock concentration in MCF-7 and MDA-MB-231 cells according to manufacturer’s instructions (QIAGEN, Hilden, Germany). Cells were transfected by FuGENE HD Transfection Reagent (Promega, Madison, WI, USA).

### 2.3. MTT Cell Viability Assay

MCF-7 and MDA-MB-231 breast cancer cells were seeded at 1 × 10^4^ density in 96-well plates (TPP Zellkultur Testplatte, Trasadingen, Sweden) and treated with E2 or FUL in a time or dose-dependent manner. Then, cells were exposed to 10 µL of 3-(4,5-dimethylthiazol-2yl)-2,5-diphenyl tetrazolium bromide dye (Sigma Aldrich, St Louis, MO, USA; final concentration: 5 mg/mL) and were incubated at 37 ℃ (Heraeus, Hera Cell 150; ThermoFisher, Waltham, MA, USA,) for 4 h for the conversion of MTT to MTT-formazan crystals by mitochondrial enzymes. After that, 100 µL DMSO (Sigma-Aldrich, St Louis, MO, USA) was added to cells for solubilization of formazan crystals. Absorbance was determined at 570 nm spectrophotometrically (Model 680 Microplate Reader Bio-Rad, Hercules, CA, USA).

### 2.4. Trypan Blue Dye Exclusion Assay-Survival Assay

Cells were seeded at 1 × 10^5^ density in 6-well plates (TPP Zellkultur Testplatte, Trasadingen, Switzerland) and treated with E2 or FUL in a time or dose-dependent manner. Then, cells were trypsinized (Trypsin EDTA (0.25%), Gibco-Life Technologies, New York, NY, USA) and were centrifuged. Cells were exposed to 0.4% (*w/v*) trypan blue (Gibco-Life Technologies, New York, NY, USA) (50 µL) and RPMI (50µL) at a 1:1 ratio. After that, 10 µL of cells were counted by a dual-chamber 0.1 mm deep Neubauer improved hemocytometer (Marienfield Superior, Lauda-Königshofen, Germany). Viable and non-viable cells are recorded, and based on viable cells a graph is formed.

### 2.5. Colony Formation Assay

The cells were seeded at a density of 3 × 10^3^ cells/well in 6-well plates and dispersed evenly by shaking the dishes slightly, and allowed to adhere for 24 h. After attachment, the MCF-7 cells were treated with E2 or FUL in a dose-dependent manner. To investigate the effect of miR-33a in E2 or FUL, mimic or anti-miR-33a transfected MCF-7 cells were treated with 100 nM E2 and 500 nM FUL. Following 24 h, drug-containing media were removed, and cells were allowed to form colonies in complete media for 10 days. The colonies were fixed with a solution of acetic acid and methanol (1:3) for 5 min. The supernatant was removed. Later, the cells were stained with 0.5% crystal violet for 30 min. Finally, the dye was washed away with distilled water. The clones were counted under a light microscope.

### 2.6. Immunoblotting Analysis

Cells were lysed on ice using ProteoJET mammalian cell lysis reagent (Fermentas, Maryland, MD, USA) containing protease inhibitors (Roche, Mannheim, Germany). Cell lysates were centrifuged at rpm for 20 min at 4 °C, and protein concentration was measured by Bradford Assay (Bio-Rad, Hercules, CA, USA). After the separation of denatured proteins according to size by SDS-PAGE, proteins are transferred to a PVDF membrane where they are labeled using antibodies specific to the target proteins. Gels obtained from SDS-PAGE were placed in the Trans-Blot Turbo transfer system (Bio-Rad, Hercules, CA, USA) using Trans-Blot^®^ turbo midi nitrocellulose transfer packs, then protein transfers were carried out at 25 V for 8 min. Membranes were blocked in 5% BSA/TBS-T (TBS containing 0.1% Tween20) for 2 h at room temperature, and then incubated with primary antibodies (Fatty Acid and Lipid Metabolism Antibody Sampler Kit #8335, PPARγ Regulated Fatty Acid Metabolism Antibody Sampler Kit #8660, Adipogenesis Marker Antibody Sampler Kit #, Nuclear Receptor Antibody Sampler Kit #8595, Autophagy Antibody Sampler Kit #4445, ULK1 Antibody Sampler Kit #8359 anti-SREBP-1, SREBP-2, FASN, HMGCR, ER, Cyclin D1, β-actin ve and β2-microglobulin (β2M), overnight at 4 °C, and later with anti-mouse or anti-rabbit IgG-HRP secondary antibody overnight at 4 °C (Cell Signaling Technology (CST), Danvers, MA, USA). After washing with TBS-Tween 20, proteins are detected using Super Signal West Femto Luminol/Enhancer Solution (ThermoScientific, San Jose, CA, USA) and exposed to Hyperfilm-ECL (Amersham Pharmacia Biotech, Piscataway, NJ, USA).

### 2.7. RNA Isolation/Quantitative Real-Time PCR

Total RNA was extracted from MCF- and MDA-MB-231 cells using miRNeasy Kit (QIAGEN, Hilden, Germany) according to the manufacturer’s protocol. Reverse transcription and cDNA synthesis were performed according to the miScript II RT Kit’s instructions (QIAGEN, Hilden, Germany). The relative expression of miR-33a was measured by CFX-Connect (Bio-Rad, Hercules, CA, USA) using miScript SYBR Green PCR Kit according to the manufacturer’s protocol (QIAGEN, Hilden, Germany). Primers for miR-33a were purchased from miScript Primer Assays (QIAGEN, Hilden, Germany). The levels of miRNA were normalized to U6 levels, respectively. The 2-CT method was used to determine the relative gene expression.

### 2.8. Statistical Analysis

Statistical analysis of experiments was performed using GraphPad Prism version 8.0.0, GraphPad Software, San Diego, CA, USA. (Available online: www.graphpad.com, accessed on 5 October 2021). MTT cell viability, cell survival assay, and PCR experiments were analyzed by one-way ANOVA followed by Tukey’s multiple comparisons test. All the experiments were repeated at least three times and significant differences were considered as *p* ≤ 0.053.

## 3. Results

In this study, we investigated the role of miR-33a in breast cancer cells, depending on the ER expression levels. We first tested the effects of E2 or FUL, a drug that is in use in clinics to treat hormone-dependent breast cancer routinely. Following incubating cells with either E2 or FUL, expression level of miR-33a significantly increased in MCF-7 cells while no significant difference was noted in MDA-MB-231 cells. The lipid metabolism signaling pathway markers were expressed differently in both cell lines which was found to be correlated with miR-33a expression levels. We finally showed that the miR-33a expression is significantly associated with AMPKα activation.

### 3.1. E2 or FUL Differently Modulated Cell Proliferation of MCF-7 Cells

**Figure 1:**
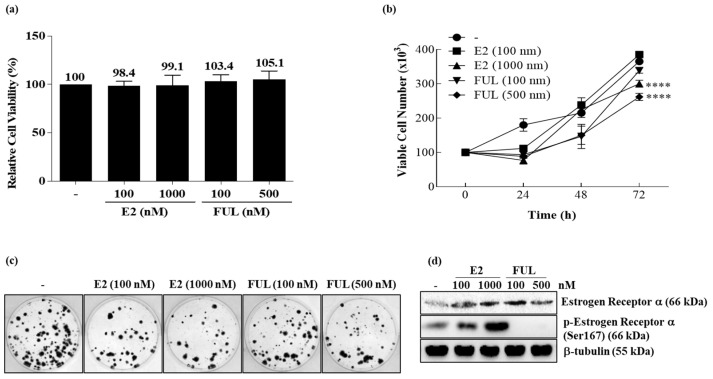
Determining appropriate concentrations of 17-β-Estradiol (E2)or Fulvestrant (FUL) treatment in MCF-7 breast cancer cells. (**a**) MTT cell viability assay proceeded to evaluate cytotoxic responses against two different concentrations of each drug. Columns represent the mean ± Std.dev of three independent experiments with at least four repeats. The analysis was performed by one-way ANOVA, Tukey’s multiple comparison test. (**b**) Viable cell numbers of MCF-7 cells treated with 100 nM and 1000 nM 17-β-Estradiol (E2), and 100 nM and 500 nM Fulvestrant (FUL) (**** *p* < 0.0001 by two-way ANOVA, Tukey’s multiple comparison test). (**c**) Representative image of colony formation assay to compare untreated control samples to 17-β-Estradiol (E2) (100–1000 nM) or Fulvestrant (FUL) (100–500 nM); (**d**) confirmation assay to evaluate E2 and FUL treatment by immunoblotting of dose-dependent E2 or FUL treated MCF-7 cell lysates. β-tubulin was used as a loading control. The relative densitometry analysis was performed by two-way ANOVA Tukey’s multiple comparison test (**** *p* < 0.0001, uncropped Western Blot Figures are shown in Figure S1).

We first studied the effects of E2 or FUL on cellular viability in MCF-7 cells. In Figure 1a, the MTT cell viability assay showed that dose-dependent E2 and FUL treatment did not cause significant cell viability in MCF-7 cells within 24 h (Figure 1a). Low concentration (100 nM) of E2 did not affect MCF-7 cell proliferation while a higher concentration of E2 has a suppressive role in MCF-7 cell proliferation. This suppression is also shown in the 500 nM FUL treatment (Figure 1b). Clonogenic formation of MCF-7 cells showed a significant decrease in response to 1000 nM E2 and 500 nM FUL treatment, which confirmed the decrease in cell viability (Figure 1c). The expression profile of ERα was investigated in MCF-7 cells and found to be upregulated in a dose-dependent manner in response to E2 treatment while downregulated in a dose-dependent manner in response to FUL treatment in MCF-7 cells (Figure 1d). Based on these results, 100 nM E2 and 500 nM FUL were used for further experiments in both MCF-7 and MDA-MB-231 cells.

### 3.2. Expression of miR-33a Was Affected by E2 or FUL Treatment in MCF-7 Cells, but Not in MDA-MB-231 Cells

**Figure 2:**
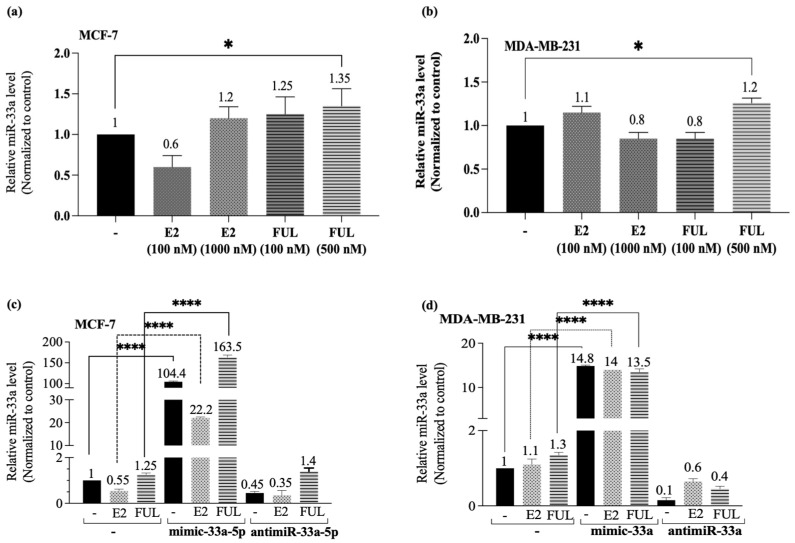
The expression profile of miR-33a, which was modulated by mimic and anti-miR transfections, in 17-β-Estradiol (E2) or Fulvestrant (FUL) treated MCF-7 and MDA-MB-231 cells. The relative miR-33a expression in 17-β-Estradiol (E2) and Fulvestrant (FUL) treated MCF-7 (**a**) and MDA-MB-231 (**b**) cells were detected by the qRT-PCR assay. Columns represent the mean ± Std.dev of three independent experiments with two repeats (* *p* = 0.0491 in MCF-7 cells, * *p* = 0.0381 in MDA-MB-231, by one-way ANOVA, Dunnett’s multiple comparison test). The mimic and anti-miR transfections changed the expression of miR-33a in MCF-7 cells (**c**) and in MDA-MB-231 cells (**d**). Columns represent the mean ± Std.dev of three independent experiments with two repeats (**** *p* < 0.0001 by two-way ANOVA, Tukey’s multiple comparison test).

To understand a putative network between fatty acid metabolism and breast cancer through determining the functional role of miR-33a, the relative expression profile of miR-33a was investigated in the presence of E2 or FUL treatment in both ER-positive MCF-7 and triple-negative MDA-MB-231 cells. A low concentration of E2 (100 nM) decreased the expression of miR-33a, while a higher concentration (1000 nM) significantly increased miR-33a expression in MCF-7 cells. However, dose-dependent FUL treatment enhanced miR-33a expression in MCF-7 cells (Figure 2a). We also investigated the miR-33a levels in triple-negative MDA-MB-231 cells and found that dose-dependent E2 did not cause a significant change in miR-33a expression, but 500 nM FUL treatment slightly increased the miR-33a expression in MDA-MB-231 cells (Figure 2b). To investigate the function of miR-33a in hormone-dependent therapies in breast cancer, both MCF-7 and MDA-MB-231 cells were transfected with syn-miR-33a (mimic-33a) or anti-miR-33a in the presence of E2 or FUL. The qRT-PCR analysis showed that enhancement of miR-33a significantly increased its expression in both E2 and FUL treatment compared to the untransfected cells; however, combined treatment of FUL with mimic-33a showed the highest expression profile in MCF-7 cells. In addition, anti-miR-33a caused a significant decrease in the miR-33a level in MCF-7 cells (Figure 2c).

Treatment of cells with mimic-33a increased its expression in MDA-MB-231 cells in the presence of both E2 and FUL. We suggested that due to the ERα−/ERβ+ characteristics of MDA-MB-231 cells, E2 or FUL treatment did not affect the miR-33a level directly. Similarly, anti-miR-33a significantly decreased miR-33a expression both in E2 and FUL treatment (Figure 2d).

### 3.3. E2 Reversed miR-33a-Mediated Energy Starvation Conditions

**Figure 3:**
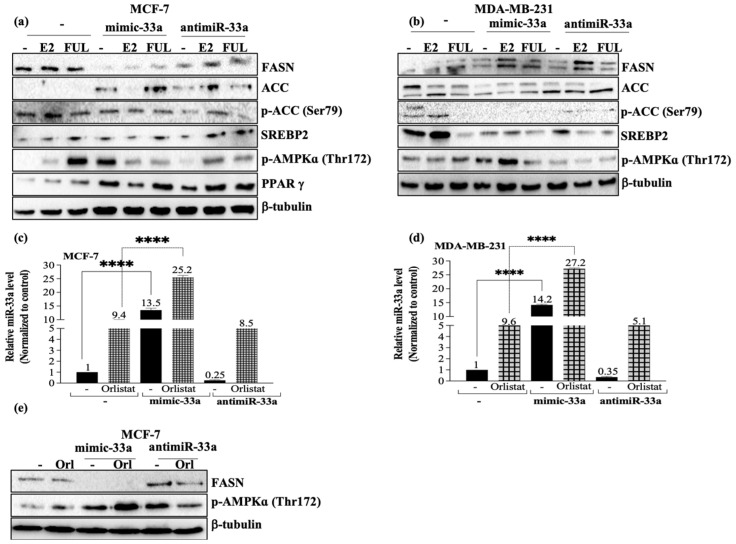
Investigation of the effect of miR-33a on fatty acid metabolism in MCF-7 and MDA-MB-231 cells. The function of miR-33a expression on fatty acid and energy metabolism in MCF-7 (**a**) and MDA-MB-231 cells (**b**) was determined by immunoblotting assay. β-tubulin was used as a loading control. The relative densitometry analysis was performed by two-way ANOVA Tukey’s multiple comparison test (**** *p* < 0.0001). The miR-33a expression was investigated by evaluating miR-33a in orlistat-treated MCF-7 (**c**) and MDA-MB-231 (**d**) cells. Columns represent the mean ± Std.dev of three independent experiments with two repeats (**** *p* < 0.0001 by two-way ANOVA, Tukey’s multiple comparison test). (**e**) FASN and p-AMPKα expression in miR-33a overexpressed/suppressed-orlistat-treated MCF-7 cells was investigated by immunoblotting assay. β-tubulin was used as a loading control. The relative densitometry analysis was performed by two-way ANOVA Tukey’s multiple comparison test (**** *p* < 0.0001). Uncropped Western Blot Figures are shown in Figure S2.

We then investigated the expression levels of FASN and acetyl-CoA carboxylase (ACC), fatty acid, and lipid metabolism signaling pathway markers, and found that MCF-7 and MDA-MB-231 cells showed different expression patterns with different estrogen levels related to miR-33a expression (Figure 3). E2 or FUL treatment did not alter FASN levels in MCF-7 cells (Figure 3a) but upregulated FASN expression in hormone unresponsive MDA-MB-231 breast cancer cells (Figure 3b). miR-33a mimics transfection to increase miR-33a levels in the cells downregulated by FASN expression in MCF-7 (Figure 3a) (** *p* = 0.0011). However, we detected the most notable upregulation for FASN in MDA-MB-231 cells following E2 or FUL treatment with miR-33a inhibitors (Figure 3b). While E2 or FUL co-treatment with miR-33a led to FASN upregulation in MCF-7 cells, FUL co-treatment was not effective to alter FASN expression levels in MDA-MB-231 cells. miR-33a expression levels in the cells are critical to adjusting FASN expression levels in hormone-responsive MCF-7 cells. When we compared miR-33a inhibitor and mimic conditions with E2 or FUL co-treatment, miR-33a inhibitor suppressed FASN upregulation in MCF-7 cells. Thus, we concluded that miR-33a is critically important in the FASN expression profile in the cells. Whereas MDA-MB-231 cells were not responsive against miR-33a expressional alteration status in the cells. In a similar way, ACC is upregulated following miR-33a mimic treatment in MCF-7 cells compared to untreated samples. While E2 co-treatment with miR-33a led to a dramatic decrease in ACC expression levels, anti-miR-33a treatment reversed this effect following E2 treatment in MCF-7 cells (Figure 3a). These alterations were also confirmed by phospho-ACC (Ser79) levels following E2 treatment. Alone, E2 treatment increased phospho-ACC levels. miR-33a inhibitor co-treatment with E2 further increased phospho-ACC levels in MCF-7 cells. These results confirmed that miR-33a suppressed activation of lipogenesis pathways in MCF-7 cells and led to the activation of AMPKα due to energy starvation conditions in MCF-7 cells. While E2 or FUL co-treatment with miR-33a mimic inhibited AMPKα activation, these drugs led to an increase in AMPKα activation status in miR-33a inhibitor treatment conditions. A similar expression profile with ACC was determined for PPARγ, which showed that miR-33a promoted de novo lipogenesis in the cells (Figure 3a).

E2 or FUL treatment led to the upregulation of FASN expression levels in MDA-MB-231 cells. miR-33a mimic treatment with E2, and FUL increased FASN expression levels. E2 co-treatment with miR-33a inhibitor treatment led to FASN upregulation, too. Thus, we concluded that FASN upregulation due to E2 treatment was regardless of miR-33a levels. On the contrary, FUL treatment was dependent on miR-33a levels while it is triggering FASN upregulation. ACC expression levels were not significantly altered following E2 or FUL treatment in MDA-MB-231 cells. Additionally, miR-33a mimic treatment led to the downregulation of ACC. While the expression levels of SREBP2 were diminished in MDA-MB-231 cells, AMPKα activation was apparent following miR-33a mimic treatment. On the contrary, miR-33a inhibitor treatment prevented AMPKα activation in MDA-MB-231 cells (Figure 3b).

In order to evaluate miR-33a-dependent regulation of lipid biogenesis, we treated cells with orlistat, which is known as a FASN inhibitor [19]. Orlistat treatment (20 µM) led to a dramatic increase in miR-33a expression profiles in MCF-7 and MDA-MB-231 breast cancer cells (Figure 3c,d). While miR-33a mimic transfection with Orlistat for 24 h further increased miR-33a expression profile, miR-33a inhibitor treatment prevented orlistat-mediated miR-33a upregulation (Figure 3c,d). FASN inhibition via orlistat treatment increased AMPKα activation. miR-33a mimic co-treatment with orlistat led to a further increase in AMPKα activation in MCF-7 cells. Thus, we concluded that miR-33a is a significant factor to increase energy starvation conditions and led to a dramatic increase in AMPKα phosphorylation levels at Thr 172 residue (Figure 3e). Additionally, altered miR-33 levels in the cells triggered deregulation of fatty acid synthesis pathways and modulated cellular energetics in breast cancer cells.

## 4. Discussion

Breast cancer is the most common cancer among women worldwide [20], while therapies are still relatively limited and therefore the need for novel therapeutic targets is of pivotal importance. Although the five-year post-treatment survival rate is high, not all respond to therapy, and some relapse after treatment. miRNAs have gained increased attention as oncogenic targets and can be located both intra- and intergenically, and can be transcribed independently from their own promoter or the promoter of the gene in which they reside [12].

Previous studies showed that MCF-7 cells are ER-positive for ERβ1, ERβ2, and ERβ5 however, the ERβ positive but ERα negative MDA-MB-231 cells are positive for ERβ1 and ERβ2 [21,22]. In addition, MCF-7 cells contain the 66 kDa ERα (ERα66) and its splice variants ERα36 and ERα46, while the MDA-MB-231 cells only contain ERα36. The variability of these ER isoforms may result in functional differences in response to estrogen-related therapies. For example, combined treatment of 17-β-Estradiol with genistein showed a suppressive role on cell proliferation and survival in MDA-MB-231 cells through upregulating apoptotic markers [21]. However, individual treatment of 17-β-Estradiol enhanced the tumor progression and metastasis of triple-negative MDA-MB-231 breast cancer cells [23].

Abnormal miRNA expression is associated with tumorigenesis and it has been accepted that some of these small non-coding RNAs act as tumor suppressors [6]. miR-33a is located in the 16th intron of SREBP-2 gene on chromosome 22 and can suppress epithelial-mesenchymal transition and metastasis in non-small cell lung cancer [24]. The miR-33 miRNA family is highly conserved from Drosophila to humans; miR-33a and miR-33b are both isoforms which are found in humans [25,26]. Moreover, miR-33a is the only miR-33 isoform that is expressed in mice and conserved in humans. Human miR-33a features two subtypes, miR-33a-3p and miR-33a-5p, which correspond to miR-33-3p and miR-33-5p in mice, respectively [27]. According to recent reports obtained from the TCGA database, miR-33 was found to be a strong predictive target for endometrial metastasis to ovarian or colorectal tissues, which was triggered by a high fat diet [28]. In a similar way, TCGA datasets showed that miR-33b differential expression was indicative for bladder cancer patients [29]. In breast cancer, diminished levels of miR-33a in malignant tissues were associated with lymph node metastasis, and the expression of miR-33a was significantly reduced in metastatic breast cancer cell lines when compared with nonmetastatic cancer cell lines and/or normal breast epithelial cells [30]. It was also reported that miR-33a enhances apoptosis and inhibits cell proliferation and metastasis in MCF-7 cells [15]. The estrogen and the ERs are well-known regulators of several aspects of glucose and lipid metabolism. In the current study, we found that more metastatic and estrogen-independent MDA-MB-231 cells express high levels of miR-33a when compared to estrogen sensitive MCF-7 cells. Estradiol treatment in ovariectomized rats has shown to lead to dramatic increase in hepatic expression of miR-33a and to cause alterations in the gene expression and the protein level of their targets, such as sterol regulatory element-binding proteins-1c (SREBP-1c), FASN, high mobility group (HMG), box transcription factor 1 (HBP1), and Sirtuin 1 (SIRT1), receptively [31]. In addition, FUL treatment as an ER blocker reversed these effects. Therefore, miR-33a acts as a tumor suppressor in breast cancer; however, its functions in general and expression profiles on triple-negative breast cancer is unclear. In our current study, we showed that a higher concentration of E2 enhanced the level of miR-33a in MCF-7 cells. The studies on the survival-related functions of E2 in MCF-7 cells demonstrated that a higher concentration of E2 upregulated the expression of ERa while the cell proliferation rate and colony-forming potentials were decreased in 1000 nM E2 treatment in MCF-7 cells. The suppressive role of higher concentration (1000 nM) of E2 was also confirmed with the relative expression profile of miR-33a in our current study. It was also found that miR-33a is a key regulator of cholesterol homeostasis as it can regulate cholesterol transporters [6]. miR-33a can regulate both liver HDL biosynthesis and cellular cholesterol efflux [32]. A previous study focusing on inflammatory breast cancer cells demonstrated that low miR-33a expression correlates with reduced cholesterol levels in cellular membranes after treating the cells with HDL [6]. On the contrary, high expression of miR-33a with increased cellular membrane following the HDL treatment was reported in non-inflammatory breast cancer cells [33]. Interestingly, the inflammatory breast cancer cells could maintain intracellular cholesterol in lipid-depleted environments [34]. It was also noted that high cholesterol levels in the cellular membrane enhance signaling pathways that affect DNA repair mechanisms that lead to radiation resistance in cancer [35]. We have found in this current study that the lipid metabolism signaling pathway markers such as FASN and ACC were differentially expressed in MCF-7 and MDA-MB-231 cells which correlated with miR-33a levels.

Recent studies indicated that miR-33a has novel roles on mitochondrial biogenesis via inhibiting AMPKα and PGC1a. It was established that the efflux of cholesterol through ABCA1 and ABCG1 requires high levels of ATP in the cells [36]. AMPKα-activation-mediated depletion of ATP cellular pool capacity can impair cholesterol efflux. When AMP/ATP ratio is increased, AMPKα activation leads to induction of a number of cellular changes including activation of PGC1α, which promote the transcription of key genes involved in mitochondrial biogenesis via NRF1 and NRF2, and TFAM. As a result of these sequential events, mitochondrial mass, capacity and ATP production is increased [36,37] Recently, many AMPKα pathway regulatory drugs have been analyzed and a number of reports have emphasized the possible roles of abnormal AMPKα signaling pathways in the regulation of growth and survival and the development of drug resistance in metastatic breast cancer [38]. AMPKα expression was shown to correlate with TNM stage and distant metastasis, while patients with higher expression of AMPKα were reported to have overall better survival and disease-free survival [38]. In the current study, the increased levels of miR-33a activated AMPKα activation through phosphorylation of Thr172 residue in both cell lines. Although E2 or FUL inhibited miR-33a-mediated AMPKα in MCF-7 cells, E2 co-treatment with miR-33a led to a dramatic increase in AMPKα levels of MDA-MB-231 cells. In a similar trend, both breast cancer cell lines showed different expression profiles against miR-33a treatment when we assessed FASN expression levels. miR-33a mimics treatment in the presence or absence of ER activator or inhibitor downregulated FASN levels in MCF-7 cells but did not act in a similar way in MDA-MB-231 cells. In accordance with previous reports [39], miR-33a was a responder gene against E2 treatment and led to alterations in lipid metabolism. In the absence of a functional ER in MDA-MB-231 cells, miR-33a-mediated lipid metabolism control did not seem to be functional.

**Figure 4:**
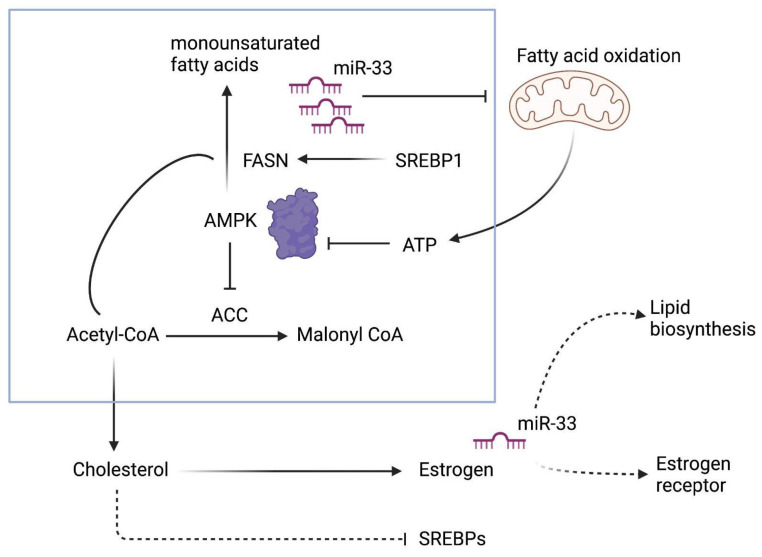
Schematic presentation of miR-33a-mediated regulation of fatty acid mechanism in the presence of estrogen receptors.

miR-33a, which is referred to as one of the critical miRNAs for cholesterol metabolism, could lead to the activation of AMPKα to increase apoptosis efficiency [40]. Therefore miR-33a expression levels in the cells might be a potential diagnostic and therapeutic marker to contribute to the link between fatty acid synthase metabolism and enhanced ER+ breast cancer development, considering the mechanistic effect on breast cancer cell survival and cell death decision provided from this study (Figure 4). Additionally, the tumor suppressor role of miR-33a in MCF-7 breast cancer development was confirmed through estrogen activation and inhibition conditions. Synthetic upregulation miR-33a might furthermore show its tumor-suppressor role through inhibiting fatty acid synthase metabolism. The interaction network of miR-33a with AMPKα could therefore be critical in further studies.

## 5. Conclusions

In the current study, we showed that the biomarker potential of miR-33a, which inhibits fatty acid synthesis, is critical in response to estrogen in breast cancer in vitro models. This suggests that cholesterol metabolism-targeting biomarkers, such as miR-33a, may be promising strategies for use in the treatment of breast cancer, also in combination with classical chemotherapeutic agents.

## Data Availability

The data presented in this study are available on request from the corresponding author.

## Supplementary Materials

The following are available online at https://www.mdpi.com/article/10.3390/cancers13215322/s1. Figure S1: Uncropped Western Blot Figures of Figure 1d: β-tubulin was used as a loading control, the relative densitometry analysis was performed by two-way ANOVA Tukey’s multiple comparison test (** *p* = 0.0022, *** *p* = 0.0003, **** *p* < 0.0001), Figure S2: Uncropped Western Blot Figures of Figure 3: β-tubulin was used as a loading control. The relative densitometry analysis was performed by two-way ANOVA Tukey’s multiple comparison test (**** *p* < 0.00001), β-tubulin was used as a loading control. The relative densitometry analysis was performed by two-way ANOVA Tukey’s multiple comparison test (** *p* = 0.057, *** *p* = 0.006, **** *p* < 0.00001).
