# Supplementary material for: In Vitro Investigations of miR-33a Expression in Estrogen Receptor-Targeting Therapies in Breast Cancer Cells
Source: Cancers (Basel). 2021 Oct 23;13(21):5322. doi: 10.3390/cancers13215322 (PMC8582455; doi:10.3390/cancers13215322)
Supplement: Supplementary file 1 [file cancers-13-05322-s001.zip › cancers-1431253-supplementary.pdf]

cancers

MDPI

Supplementary Materials: In Vitro Investigations of miR-33a Expression in Estrogen Receptor-Targeting Therapies in Breast Cancer Cells

Pelin Ozfiliz-Kilbas, Ozlem Sonmez, Pinar Obakan-Yerlikaya, Ajda Coker-Gurkan, Narcin Palavan-Unsal, Pinar Uysal-Onganer and Elif Damla Arisan

Chemiluminescence

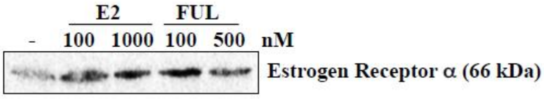

Raw image

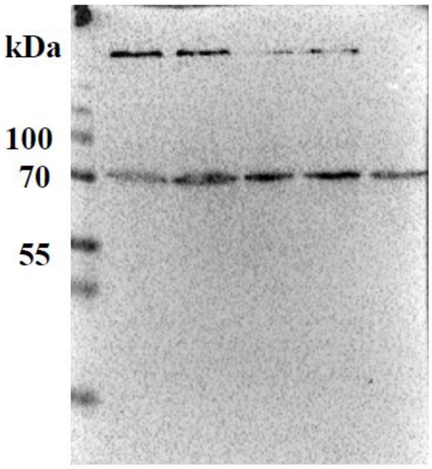

IB: Estrogen Receptor α (66 kDa)

Chemiluminescence

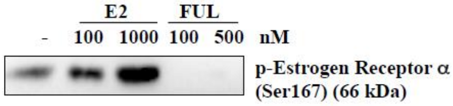

Raw image

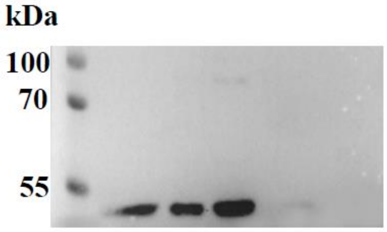

IB: Estrogen Receptor α Ser 167 (66 kDa)

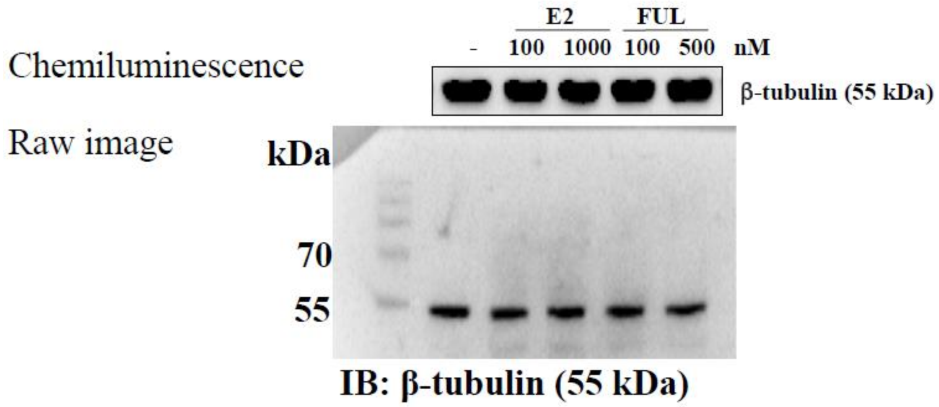

Figure S1. Uncropped Western Blot Figures of Figure 1d.

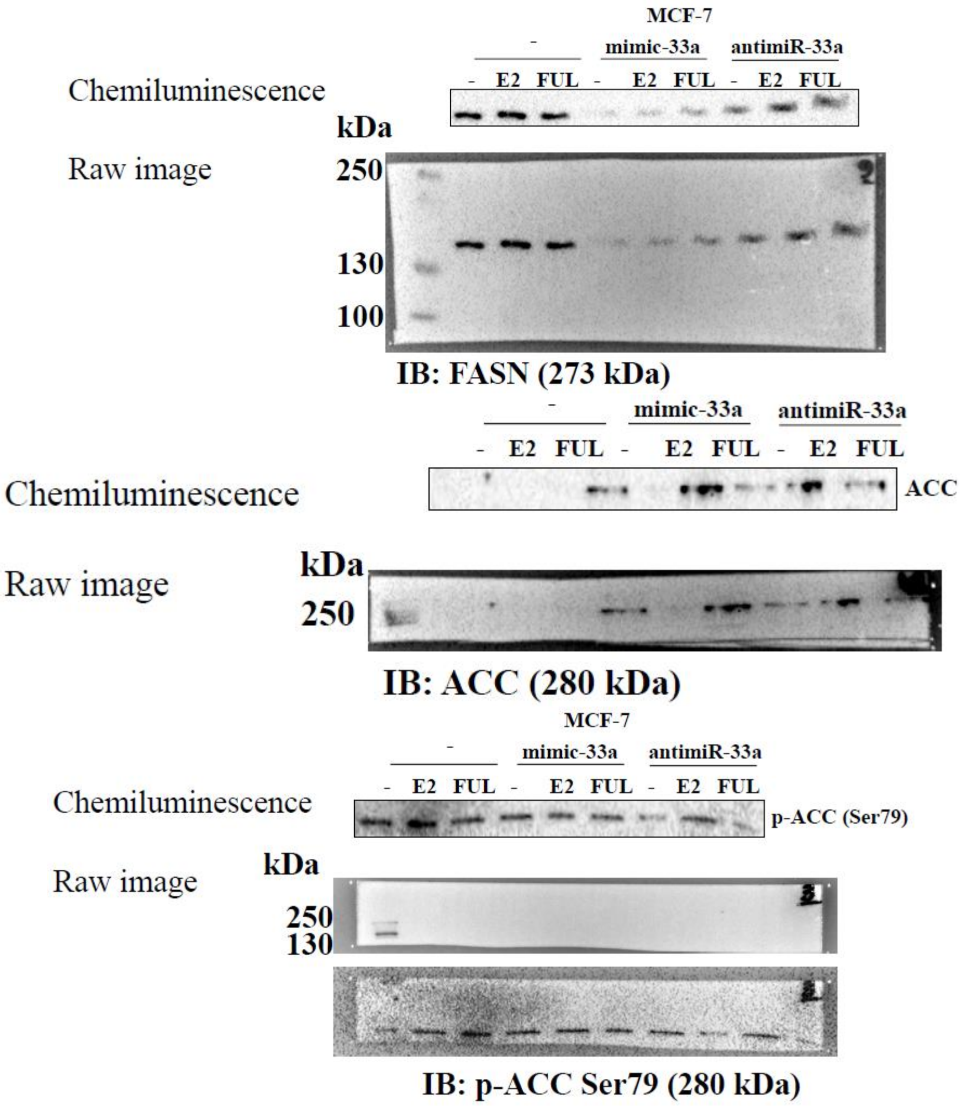

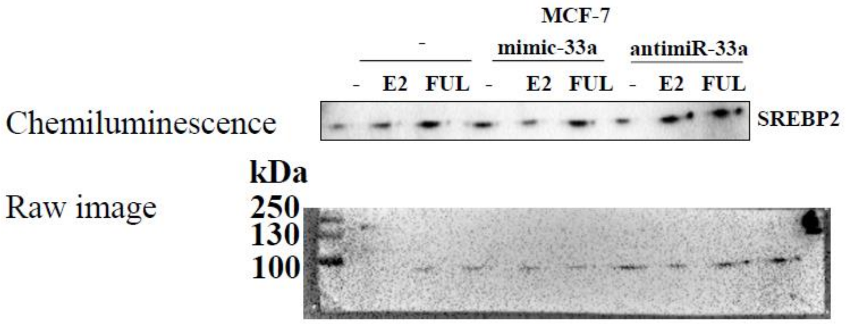

IB: SREBP2 (128 kDa)

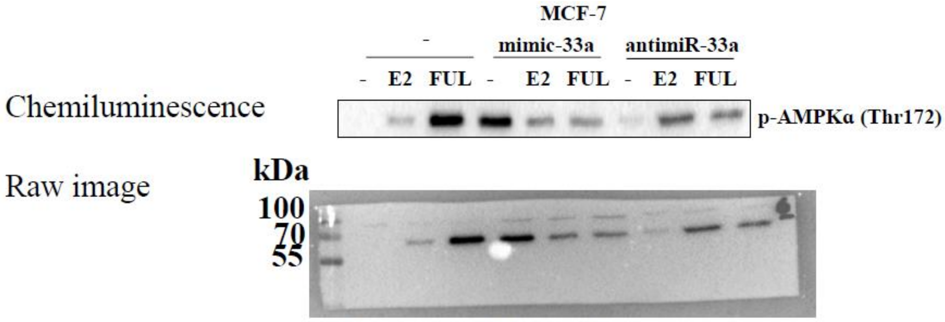

IB: p-AMPK Thr172 (62 kDa)

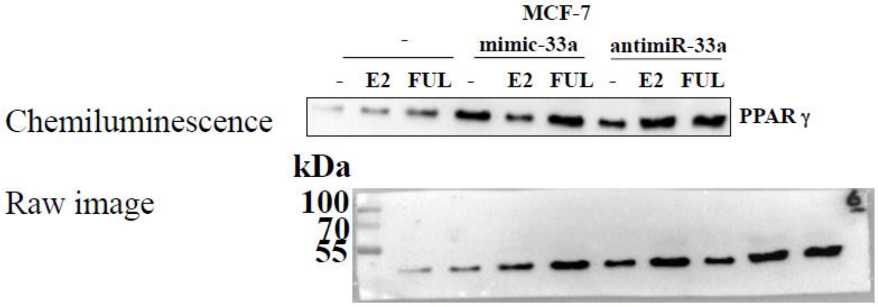

IB: PPARγ (53 kDa)

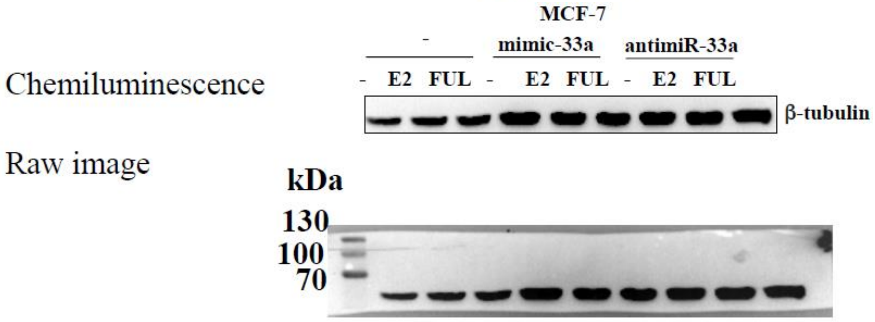

IB: β-tubulin (55 kDa)

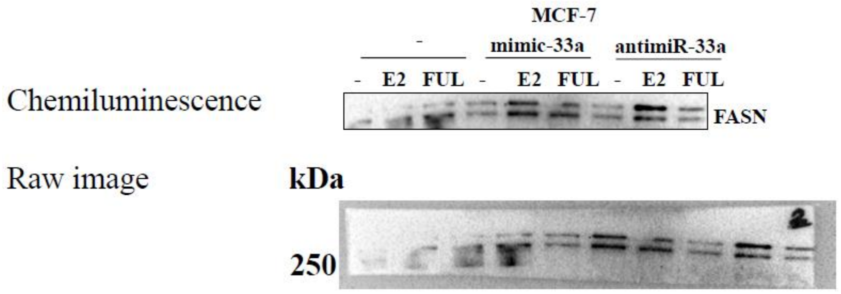

IB: FASN (273 kDa)

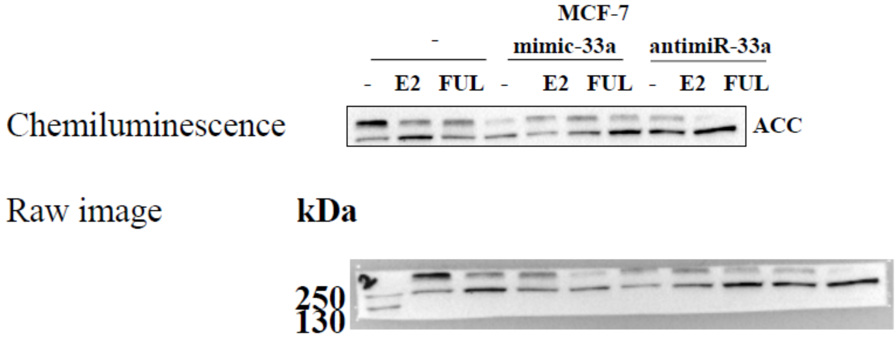

IB: ACC (280 kDa)

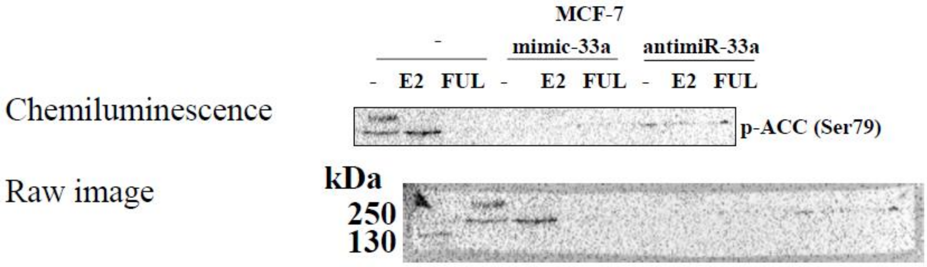

IB: p-ACC Ser79 (280 kDa)

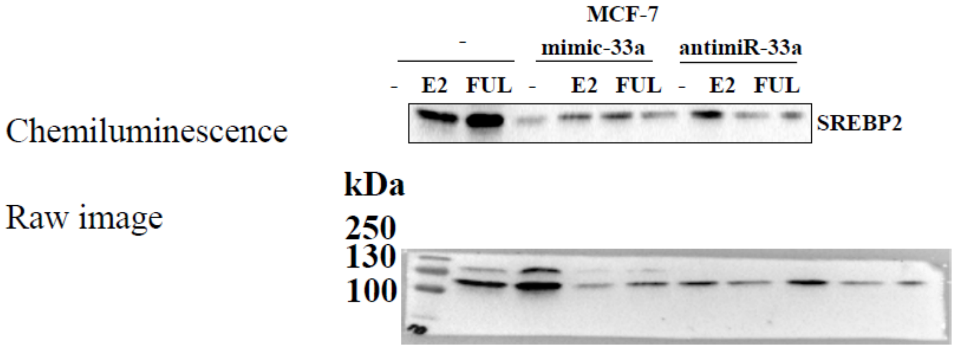

IB: SREBP2 (128 kDa)

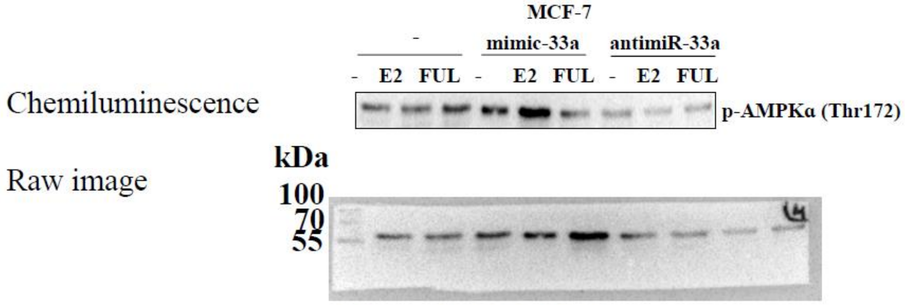

IB: p-AMPK (62 kDa)

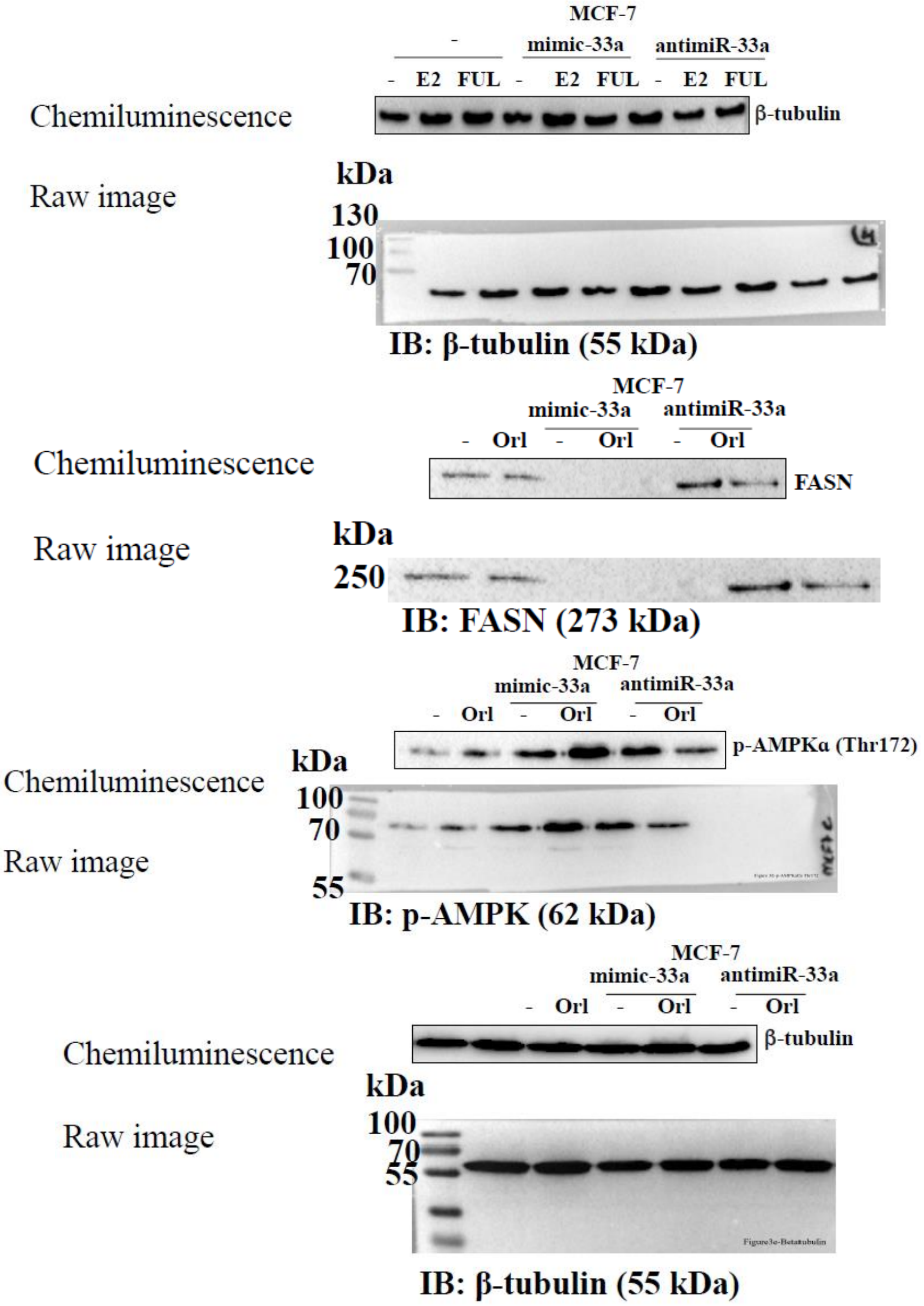

Figure S2. Uncropped Western Blot Figures of Figure 3.
